# The updated strategy to overcome new challenge: Omicron variant (B.1.1.529) pandemic

**DOI:** 10.1002/ctd2.28

**Published:** 2022-03-05

**Authors:** HyokJu Ri, Xin Chen

**Affiliations:** ^1^ The Second Hospital Dalian Medical University Dalian China; ^2^ The Hospital of Pyongyang Medical College Pyongyang DPR of Korea

**Keywords:** coronavirus, COVID‐19, Omicron variant, SARS‐CoV‐2

## Abstract

The new variant was discovered in South Africa and threatened the word of concerning new pandemic coming from this variant. The Omicron variant likely would spread more easily than the original SARS‐CoV‐2 virus, and can spread the virus to others, even if they are vaccinated or don't have symptoms.

WHO first reported the new strain on November 24, 2021, after a case was reported in South Africa in November, later calling it a ‘variant of concern’ and that early evidence ‘suggests an increased risk of reinfection’. Following the discovery of the SARS‐CoV‐2 Omicron variant (B.1.1.529), the global COVID‐19 outbreak has resurfaced after appearing to be relentlessly spreading over the past two years.

COVID‐19 has infected more than 270 million people and killed over 5.3 million worldwide. At this time, the new variant was discovered in South Africa and threatened the word of concerning new pandemic coming from this variant.^1^ WHO first reported the new strain on November 24, 2021, after a case was reported in South Africa in November, later calling it a ‘variant of concern’ and that early evidence ‘suggests an increased risk of reinfection’. Following the discovery of the SARS‐CoV‐2 Omicron variant (B.1.1.529), the global COVID‐19 outbreak has resurfaced after appearing to be relentlessly spreading over the past 2 years. This new variant showed marked degree of mutation, compared with the previous SARS‐CoV‐2 Omicron variants.^2^ The latest severe acute respiratory syndrome Omicron (B.1.1.529) variant has ushered panic responses around the world due to its contagious and vaccine escape mutations.^3^

After discovering Omicron variant, WHO designated the five ‘Variants of Concern’ including 20I (Alpha, V1) or B.1.1.7, 20H (Beta, V2) or B.1.351, 20I (Gamma, V3) or P.1, 21A (Delta) or B.1.617.2, 21K(Omicron) or B.1.1.529. Among these five variants, the new variant Omicron is more potentially transmissible than the others and prompted a fresh round of travel restrictions across the world raising the concerns about what can be the next pandemics.

There are two kinds of Omicron variants, 21K and 21L. Variant 21K(Omicron) appears to have arisen in November 2021, possibly in South Africa. Early sequences are predominantly from South Africa, though also detected in Botswana and Hong Kong. 21K(Omicron) is primarily of concern due to the large number of mutations it has in the Spike gene. Many of these variants are in the receptor binding domain and N‐terminal domain and thus may play key roles in ACE2 binding and antibody recognition.^4^, ^5^, ^6^, ^7^ 21L (also known as BA.2) is a sister‐lineage to 21K(Omicron), and both are part of Pango lineage B.1.1.529. 21L and 21K(Omicron) share 38 nucleotide and amino‐acid mutations, but 21L has an additional 27, while 21K(Omicron) has an additional 20.

Looking just at Spike, both share 21 amino‐acid mutations, with 21K(Omicron) carrying an additional 12 unique amino‐acid mutations and 21L carrying an additional 6 (plus a deletion/mutation – see below). Notably, 21L lacks the deletion at S:H69‐ and S:V70‐ (see MutationS:H69‐)) which cases the ‘S‐gene drop out’ or SGTF that has been used to track 21K(Omicron) in TaqPath PCR tests (https://www.nasdaq.com/articles/thermo‐fisher%3A‐taqpath‐covid‐19‐tests‐detect‐sars‐cov‐2‐in‐samples‐containing‐omicron). Among these two variant types, 21K is much awful than the other, and the world is suffering from this variant due to worrying about new coming pandemic. The most important point is that this variant can escape the vaccines and immune system.

A major reason is Omicron has accumulated over 50 mutations, including about 30 in the spike protein, the part of the coronavirus that mRNA vaccines teach our immune systems to attack. All of these genetic changes raise the possibility that Omicron could cause breakthrough infections in people who have already received a Pfizer or Modern mRNA vaccine (Latest on Omicron Variant and COVID‐19 Vaccine Protection – NIH Director's Blog). In lab studies working with live Omicron virus, the researchers showed that this variant still relies on the ACE2 receptor to infect human lung cells. That is really good news. It means that the therapeutic tools already developed, including vaccines, should generally remain useful for combatting this new variant.^8^

The others of great interest were the first results of the Pfizer study, which the company made available in a news release (https://www.pfizer.com/news/press‐release/press‐release‐detail/pfizer‐and‐biontech‐provide‐update‐omicron‐variant). These studies showed that the neutralising ability of samples from those who had received two shots had a more than 25‐fold decline relative to the original virus. In much more encouraging news, their studies went on to show that a booster dose of the Pfizer vaccine raised antibody levels against Omicron to a level comparable to the two‐dose regimen against the original variant. Together with the South Africa data, it suggests that getting booster shot or third dose of vaccine may be to protect against breakthrough infections with the Omicron variant, but may not be enough.

While efforts already are underway to develop an Omicron‐specific COVID‐19 vaccine, these findings suggest that it is already possible to get good protection against this new variant by getting a booster shot.

In conclusion, what will not change, though, is that vaccines are the best way to protect yourself and others against COVID‐19. Wearing a mask, especially in public indoor settings, offers good protection against the spread of all SARS‐CoV‐2 variants. And the second point is tougher restrictions would prevent thousands of deaths from Omicron.
